# Supplementary material for: Content-rich biological network constructed by mining PubMed abstracts
Source: BMC Bioinformatics. 2004 Oct 8;5:147. doi: 10.1186/1471-2105-5-147 (PMC528731; doi:10.1186/1471-2105-5-147)
Supplement: Additional File 2 — The original results of the above study (non-essential files are deleted to keep the file size under the limit set by BMC bioinformatics). [file 1471-2105-5-147-S2.bz2 › chilibotAdditionalFile2/dip20/717ID10781811E18054/html/TATA_TATB.html]

 


 **TATA** and **TATB** 
  
Found 20 abstracts in PubMed, retrieved 20.  
 

 What does Google say? 
 PDF only 
| .edu only 

---

**Interactive relationship** (e.g. stimulation, inhibition, etc)

**Neutral relationship**- Membrane interactions and self association of the  **TatA**  and  **TatB**  components of the twin arginine translocation pathway.  Ref: 11591389 FEBS Lett, 2001
- The three isolated, affinity tagged Tat  [ **TATA** ]  complexes comprised  **TatA** ,  **TatB**  and TatC in each case, demonstrating a strong interaction between these three subunits.  Ref: 12823967 J Mol Biol, 2003
- This conclusion is supported by the observation that Helicobacter pylori  **tatA**  will complement an E. coli  **tatA**  mutant, but not a  **tatB**  mutant.  Ref: 10593889 J Biol Chem, 1999
- A chimeric protein comprising the N terminal region of  **TatA**  fused to the amphipathic and C terminal domains of  **TatB**  supports a low level of Tat  [ **TATA** ]  activity in a strain in which the wild type copy of either  **tatA**  or  **tatB**  but not both is deleted.  Ref: 12374820 J Bacteriol, 2002
- The Tat  [ **TATA** ]  system involves the integral membrane proteins  **TatA** ,  **TatB** , TatC, and TatE.  Ref: 10593889 J Biol Chem, 1999
- Further, homologues most resembling  **TatB**  are more sequence divergent than those more closely resembling  **TatA** .  Ref: 12029389 Arch Microbiol, 2002
- Phylogenetic analyses reveal that most prokaryotic Tat  [ **TATA** ]  systems consist of one TatC homologue and two sequence divergent  **TatA**  homologues  **TatA**  and  **TatB** .  Ref: 12029389 Arch Microbiol, 2002
- Surprisingly, large scale mutagenesis of the conserved amphipathic regions of  **TatA**  and  **TatB**  leads only to minor effects on Tat  [ **TATA** ]  dependent export suggesting that this particular feature is not central to the translocation mechanism.  Ref: 12606028 FEBS Lett, 2003
- Its main components are  **TatA** ,  **TatB**  and TatC, all of which are required for Tat  [ **TATA** ]  dependent export.  Ref: 12773183 Biochem Soc Trans, 2003
- When only one  **TatA**  homologue is present,  **TatB**  is missing, and when three  **TatA**  homologues are present, the third one arose by duplication of  **TatA** , not  **TatB** .  Ref: 12029389 Arch Microbiol, 2002
- Proteins bearing a signal peptide with a consensus twin arginine motif are translocated via the Tat  [ **TATA** ]  pathway, a multiprotein system consisting minimally of the integral inner membrane proteins  **TatA** ,  **TatB** , and TatC.  Ref: 12427031 Biochemistry, 2002
- Our data suggest that the  **TatA** ,  **TatB**  and TatC proteins are found in at least two major types of high molecular mass complex in detergent solution, one consisting predominantly of  **TatA**  but with a small quantity of  **TatB** , and the other based on a TatBC unit but also containing some  **TatA**  protein.  Ref: 12367533 J Mol Biol, 2002
- The genes  **tatA** ,  **tatB** , tatC and tatE code for integral membrane proteins that are components of the Tat  [ **TATA** ]  pathway.  Ref: 11422364 Eur J Biochem, 2001
- Deletion of the  **tatB**  gene alone is sufficient to block the export of seven endogenous Tat  [ **TATA** ]  substrates, including hydrogenase 2.  Ref: 10593889 J Biol Chem, 1999
- Cells co overexpressing tatABCDE show an increased rate of export of a signal peptide defective Tat  [ **TATA** ]  precursor protein and a complex containing the  **TatA**  and  **TatB**  proteins can be purified from the membranes of such cells.  Ref: 11422364 Eur J Biochem, 2001

**Non-interactive relationship** (e.g. studied together, co-existance, homology, etc.)

- In the present study, we have purified the Tat  [ **TATA** ]  complex from E. coli, and we show that it contains only  **TatA** ,  **TatB** , and TatC.  Ref: 11279240 J Biol Chem, 2001
- Using co immunoprecipitation experiments, we show that  **TatA**  and  **TatB**  do indeed associate with each other.  Ref: 10781811 FEBS Lett, 2000
- These results imply that the transmembrane and amphipathic helical regions of  **TatA**  and  **TatB**  are critical for their function but that the C terminal domains are not essential for Tat  [ **TATA** ]  transport activity.  Ref: 12374820 J Bacteriol, 2002
- The  **TatA**  and  **TatB**  proteins are essential components of the twin arginine protein translocation pathway in Escherichia coli.  Ref: 12374820 J Bacteriol, 2002
- Four insertions analyzed had no effect on the stability of the altered TatC proteins or on membrane assembly of the  **TatA**  and  **TatB**  proteins.  Ref: 12163163 FEBS Lett, 2002
- Of these,  **TatA** ,  **TatB** , and TatE are homologues of the Hcf106 component of the DeltapH dependent protein import system of plant thylakoids.  Ref: 10593889 J Biol Chem, 1999
- Gel filtration chromatography demonstrates that both proteins are present in a large complex with an apparent molecular mass of approximately 600 kDa, indicating the presence of other components and or several  **TatA**  and  **TatB**  subunits.  Ref: 10781811 FEBS Lett, 2000
- Here is the report of knocked out the  **tatA** ,  **tatB**  and tatC genes of the V. cholerae by suicide plasmid homologous recombination technology.  Ref: 12561481 Yi Chuan Xue Bao, 2002
- The first region is the interdomain hinge between the transmembrane and the amphipathic alpha helices of  **TatA**  and  **TatB**  proteins.  Ref: 12650927 FEBS Lett, 2003
- Analysis of Tat  [ **TATA** ]  component stability in various tat  [ **TATA** ]  deletion backgrounds shows that TatC is rapidly degraded in the absence of  **TatB**  suggesting that TatC complexes, and is stabilized by,  **TatB** .  Ref: 10593889 J Biol Chem, 1999
- In this report we have studied the functions of conserved residues within the structurally related  **TatA**  and  **TatB**  proteins.  Ref: 12650927 FEBS Lett, 2003
- The second region is within the amphipathic helices of  **TatA**  and  **TatB** .  Ref: 12650927 FEBS Lett, 2003
- Co immunoprecipitation experiments show that TatC is required for the interaction of  **TatA**  with  **TatB** , suggesting that  **TatA**  may interact with the complex via binding to TatC.  Ref: 11279240 J Biol Chem, 2001
- The essential Tat  [ **TATA** ]  pathway components  **TatA** ,  **TatB**  and TatC are shown to be integral membrane proteins.  Ref: 11591389 FEBS Lett, 2001
- The integral membrane proteins  **TatA** ,  **TatB**  and TatC are essential components of this pathway.  Ref: 12367533 J Mol Biol, 2002
- The Escherichia coli twin arginine translocase conserved residues of  **TatA**  and  **TatB**  family components involved in protein transport.  Ref: 12650927 FEBS Lett, 2003
- A similar DeltapH export system of Escherichia coli contains four components, twin arginine translocation A  **TatA** ,  **TatB** , TatC, and TatE.  Ref: 11526245 Proc Natl Acad Sci U S A, 2001
- The maize tha4 gene functions in sec independent protein transport in chloroplasts and is related to hcf106,  **tatA** , and  **tatB** .  Ref: 10525534 J Cell Biol, 1999
- Truncation analysis of  **TatA**  and  **TatB**  defines the minimal functional units required for protein translocation.  Ref: 12374820 J Bacteriol, 2002
- The sequence of the tha4 gene product resembles those of the maize hcf106 gene and the bacterial  **tatA**  and  **tatB**  genes.  Ref: 10525534 J Cell Biol, 1999
- Sequence comparisons suggest that tha4 more closely resembles  **tatA** , and hcf106 more closely resembles  **tatB** .  Ref: 10525534 J Cell Biol, 1999
- Finally, we show that  **TatA**  is stable in the absence of  **TatB**  and may participate in a separate complex lacking  **TatB**  in wild type cells.  Ref: 10781811 FEBS Lett, 2000
- The  **tatA**  and  **tatB**  genes encode important components of the export system and we have analysed whether the proteins encoded by these genes physically interact.  Ref: 10781811 FEBS Lett, 2000
- In addition to TatE and TatC proteins, either  **TatA**  or  **TatB**  was sufficient for the translocation of RR ColV or KR ColV.  Ref: 11922668 J Mol Biol, 2002
- Genetic studies revealed that one maize protein involved in this mechanism, HCF106, is related in both structure and function to the bacterial  **tatA**  and  **tatB**  gene products.  Ref: 10525534 J Cell Biol, 1999
- In contrast,  **TatA**  plus the conserved N terminal domain of  **TatB**  were required to mediate the killing effect of ColV fused to the less efficient RK signal peptide.  Ref: 11922668 J Mol Biol, 2002
- The purified TatAB complex has an apparent molecular mass of 600 kDa as measured by gel permeation chromatography and, like the membranes of wild type cells, contains a large molar excess of  **TatA**  over  **TatB** .  Ref: 11422364 Eur J Biochem, 2001
- Complementation analysis indicates that while  **TatA**  and TatE are functionally interchangeable, the  **TatB**  protein is functionally distinct.  Ref: 10593889 J Biol Chem, 1999
- The translocation of the RR GFP fusion required  **TatA** ,  **TatB**  and TatC proteins.  Ref: 11922668 J Mol Biol, 2002
- Genes known to be involved in this process include  **tatA** ,  **tatB** , and tatC that form an operon with a fourth gene, tatD.  Ref: 10747959 J Biol Chem, 2000
- Chemical crosslinking studies indicate that  **TatA**  forms at least homotrimers, and  **TatB**  minimally homodimers, in the native membrane environment.  Ref: 11591389 FEBS Lett, 2001
- This Tat  [ **TATA** ]  BC chimera supports efficient Tat  [ **TATA** ]  dependent export, indicating that  **TatB**  and TatC act as a unit in both structural and functional terms.  Ref: 11279240 J Biol Chem, 2001
